# Association between antihypertensive medication use and kidney cancer risk: a meta-analysis accounting for hypertension

**DOI:** 10.1186/s12885-025-14406-3

**Published:** 2025-06-06

**Authors:** Minji Jung, Mingyi Li, Jaekyu Shin, Benjamin I. Chung, Marvin E. Langston

**Affiliations:** 1 Department of Urology, Stanford University Medical Center, Stanford, CA USA; 2 Department of Epidemiology and Population Health, School of Medicine, Stanford University, Stanford, CA USA; 3 Department of Clinical Pharmacy, School of Pharmacy, University of California, San Francisco, CA USA

**Keywords:** Antihypertensive drugs, Kidney cancer, Renal cell carcinoma, Hypertension, Adverse drug reaction

## Abstract

**Background:**

Evidence that antihypertensive medication (AHTN) use is associated with an increased risk of kidney cancer (KC) is emerging. However, limited evidence is available on disentangling the effects of AHTN use on KC from hypertension, which is a risk factor for KC. We aimed to identify pooled estimates for the associations between AHTN use and KC risk, independent of hypertension.

**Methods:**

We searched for observational studies that investigated the associations between AHTN use and KC through January 2025. To identify the independent effects of AHTN from hypertension, we conducted stratified analyses with and without accounting for hypertension: any methods (matching, adjustment, or stratification/restriction) versus none. We conducted random-effects meta-analyses with robust variance estimation to calculate pooled relative risk (RR).

**Results:**

In this meta-analysis consisting of 39 eligible studies, AHTN use was associated with an increased risk of KC based on estimates that accounted for hypertension (RR 1.19, 95% confidence interval (CI) 0.93–1.52 for angiotensin-converting enzyme inhibitor; RR 1.15, 95% CI 1.00-1.31 for angiotensin receptor blocker; RR 1.09, 95% CI 1.03–1.16 for beta-blocker, RR 1.40, 95% CI 1.12–1.75 for calcium channel blocker (CCB); RR 1.36, 95% CI 1.20–1.55 for diuretic; and RR 1.40, 95% CI 1.13–1.75 for non-classified AHTN). Findings from duration‒response relationships supported the main findings.

**Conclusions:**

AHTN use was associated with an increased risk of KC compared to no use, even after accounting for hypertension, with the highest risk observed for CCB. Our findings highlight the potential KC risks associated with different AHTN classes, with optimal cardiovascular care remaining an important consideration.

**Supplementary Information:**

The online version contains supplementary material available at 10.1186/s12885-025-14406-3.

## Background

Kidney cancer (KC) was the 14th most commonly diagnosed cancer worldwide in 2022, with 434,419 new cases, accounting for 2.2% of all cancer sites [1]. The incidence of KC has constantly increased in the US (2014–2018). Among the 18 most prevalent cancers, KC was ranked third among men (average annual percent change: 0.7%) and fourth among women (average annual percent change: 1.2%) [1, 2]. Due to a lack of nationwide screening and few signs or symptoms in the early stages, many KC cases are detected at late stages, which have poor outcomes with a 5-year survival of under 20% [3]. In 2020, the national burden for KC care was estimated to be $9.7 billion, which was ranked seventh of all cancers, and it is expected to increase further [4]. 

Antihypertensive medication (AHTN) use has been shown to be effective in controlling hypertension and preventing cardiovascular diseases [5]. Hypertension is a strong risk factor for KC [6], potentially through chronic inflammation, cellular hypoxia response activation, and hypoxia-induced factor expression [7, 8]. Therefore, it is reasonable to assume that lowering high blood pressure through AHTN use may reduce KC risk. However, emerging evidence has shown the reverse results that AHTN, especially calcium channel blocker (CCB) or diuretic (DU), is associated with a higher risk of KC [9–17]. This increased risk is driven by several possible mechanisms such as facilitating tumor cell proliferation, angiogenesis, or migration, altering intracellular calcium levels and inhibiting apoptosis, or causing renal adenomas, nephropathy, or chronic inflammation [16–18]. This suggest that AHTN use may independently increase KC risk, regardless of its blood pressure-lowering effects.

Considering potential confounding by indication from hypertension, it is challenging to disentangle the effect of AHTN use on KC from that of high blood pressure [6]. More severe hypertension often leads to increased use of the medications, which may also be associated with a higher risk of KC. Given their long-term and widespread use [5], a better understanding is necessary to increase awareness of drug safety and to develop cancer preventive strategies. This meta-analysis aimed to investigate the associations between AHTN use and KC risk, distinguishing them from associations related to hypertension.

## Method

### Study eligibility criteria

This meta-analysis included observational cohort and case-control studies, which investigated the associations between AHTN use and KC risk. AHTN included angiotensin-converting enzyme inhibitor (ACEI), angiotensin receptor blocker (ARB), beta-blocker (BB), CCB, and DU based on the American College of Cardiology/American Heart Association Guidelines [5]. Studies that reported combined results for any classes of AHTN or non-DU were grouped as “Any AHTN” in this meta-analysis. KC included total cases and renal cell carcinoma (RCC), the most common subtype (~ 90%) of KC. This meta-analysis adhered to the guidelines for conducting meta-analyses of observational studies and the Preferred Reporting Items for Systematic reviews and Meta-Analyses (PRISMA) statement (Table S1) [19]. 

### Search strategy

The published literature was searched through January 10, 2025. Comprehensive search strategies were established and implemented in PubMed, Embase, Web of Science, and Cochrane Library database. Standardized terms and key words, including AHTN, ACEI, ARB, BB, CCB, DU, hypertension, KC, and RCC (Table S2), were used during the search process. The titles and abstracts of 8,476 articles were screened to exclude reviews, commentaries, editorials, case reports, abstract-only articles, preclinical studies, original studies without exposure or outcome of interests, and original studies written in non-English languages (Figure S1). Two independent reviewers performed the systematic review with disagreements settled by a third independent reviewer.

### Analysis design and data extraction

Data extraction was completed by two independent reviewers. Any conflicts or inconsistencies were evaluated by the third reviewer. The final decision for disagreements was reached through consensus among all reviewers. Study estimates were extracted through pre-defined two steps. Firstly, we selected one estimate per AHTN class per study. We identified the estimate as the most fully adjusted estimate shown in the abstract or the main result. When studies reported separate estimates for specific subgroups (e.g., men and women [9], subtypes of DU [9], duration of medication use [20], or hypertensive and non-hypertensive populations [10, 21]), multiple estimates per AHTN per study were chosen. Secondly, to evaluate the independent effect of AHTN on KC risk from that of hypertension, we conducted stratified analyses based on the methods each contributing study used to account for hypertension: (1) statistical adjustment for or matching on hypertension, (2) stratification or restriction to hypertensive population, and (3) none. When the estimate or confidence interval (CI) were not reported but raw numbers (i.e., exposed cases, exposed non-cases, unexposed cases, and unexposed non-cases) were provided [12, 21–27], we manually calculated the crude odds ratio (OR) and 95% Wald CI using the provided standard errors on the log scale [28]. The third reviewer confirmed the calculations and compared them with crude estimates from other studies to ensure our estimates fall within the expected range. Since KC is rare, we assumed that OR approximates to risk ratios.

### Quality assessment based on the Newcastle-Ottawa Quality Assessment scale

The quality of each study was assessed using a revised version of the Newcastle-Ottawa Quality Assessment scale aligned with our study purpose [29]. The revised scale assessed the quality of studies based on three aspects: (1) whether the KC occurrence was clinically or histologically ascertained; (2) whether the studies accounted for confounders (i.e., hypertension, smoking, body mass index (BMI), age, and sex); and (3) whether individual class of AHTN was identified. A sum score of ≤ 7 and 8–10 was regarded as low to moderate-quality and high-quality, respectively. Detailed information on the scale is shown in Table S3.

### Statistical analysis

This meta-analysis used random-effects models to calculate pooled risk estimates (relative risk, RR) for the association between different classes of AHTN and KC risk, based on estimates that accounted for hypertension as well as those that did not. To investigate the possible influence of other factors on the pooled estimates and identify sources of heterogeneity, we performed subgroup analyses. These analyses included the following factors: (1) outcome types (KC and RCC); (2) AHTN use assessment (medical records and self-reported data); (3) sex; (4) publication year (before and after 2000); (5) study designs (cohort and case‒control study); (6) estimate types (hazard ratio and OR); (7) study quality (low-moderate and high); and (8) geographical region of the study population (US/Canada, Europe/Middle East, and Asia/Australia). Considering the potential influence of hypertension in the subgroup analyses, we further conducted the analyses using the estimates that accounted for hypertension when possible [30]. To assess whether the findings were consistent by BMI or smoking, which are also known as strong risk factors for KC in addition to hypertension, we summarized the pooled effectsestimates stratified by studies that accounted for hypertension only and those that further adjusted for smoking or BMI.

To evaluate the relationships between the duration of AHTN use and KC risk, we performed analyses for each class of AHTN based on estimates that accounted for hypertension [31]. We determined the value of duration as the median length of each time period and displayed the corresponding estimate in a bubble plot. Bubble plots (a scatter plot weighted by standard error) were used to show the effect size (the natural log of RR) against the exposure duration. The size of each bubble reflected the study sample size, with larger bubbles indicating larger sample size. To evaluate the impact of publication bias, we used Begg’s funnel plot and Egger’s regression test [32, 33]. To address the potential issue of overlapping populations between studies or within a study involving multiple exposures, we stratified by AHTN class and applied random-effects models with robust variance estimation (RVE). The random-effects model accounts for both between-study and within-study variances, and RVE ensures consistent standard error estimates despite within-study correlations [34, 35]. Additionally, we conducted two sensitivity analyses: (1) excluding nine overlapping studies using the same database within a similar study period [9, 10, 12, 21, 36–40] and (2) considering overlaps by AHTN class, retaining one study per class, and excluding overlapping studies with lower quality scores or smaller sample sizes, resulting in the exclusion of four studies on ARB and DU [37–40]. To determine the robustness of our results and assess the potential of underestimated heterogeneity due to highly influential studies, we also performed a leave-one-out sensitivity analysis [41]. We assessed the heterogeneity in estimates using the I^2^ statistic [42]. We defined statistical significance as a two-tailed p value < 0.05. All data analyses were conducted using R Version 4.2.2.

## Results

### Characteristics of studies

This meta-analysis included a total of 39 observational studies (Figure S1). Of these studies, 24 were cohort studies, nested case‒control studies, and case-cohort studies, and 15 were case‒control studies. AHTN use was assessed using medical records in 24 studies and self-reports/surveys in 15 studies. Studies were derived from various populations in US/Canada (*n* = 19), Europe (*n* = 10), Middle East (*n* = 1), Asia (*n* = 8), and Australia (*n* = 1). Among all included studies, 33 accounted for hypertension using adjustment or matching (*n* = 18) and stratification or restriction by hypertension status (*n* = 15). The study quality assessment scores ranged from 2 to 10 (Table 1 & Table S4).

**Table 1:** Characteristics of 39 studies that evaluated the associations between AHTN and kidney cancer incidence

First author	Publication year	Geographical region	Data collection period	Type of study	Class of AHTN	Outcome	Cases/participants	Age (year)	Methods to account for hypertension^a^	QS
Assimes, TL [54]	2008	Canada	1980–2003	NCC	ACEI/ARB, BB, CCB	KC	11,697/77,887	71.8-case; 71.7-control (mean)	A	8
Braun, S [23]	1998	Israel	1990–1993	Cohort	CCB	KC	13/11,575	45–74	N	3
Chang, PY [36]	2015	Taiwan	2000–2011	Cohort	BB	KC	26/24,238	≥ 20	A	8
Chen, LC [37]	2024	Taiwan	2001–2016	Cohort	DU	RCC	NA/17,212	≥ 20	M, A	8
Cho, IJ [38]	2021	Korea	2005–2012	Cohort	ACEI, ARB, BB, CCB, DU	KC	173/625,503	55.2 (mean)	S	10
Chow, WH [56]	1995	US	1988–1990	CC	DU, Non-DU	RCC	151/842	20–79	A	7
Chuang, YW [9]	2017	Taiwan	2005–2011	NCC	ACEI, ARB, CCB, DU	KC	8,337/32,167	≥ 20	S	8
Colt, JS [10]	2017	US	2002–2007	CC	ACEI, BB, CCB, DU	RCC	1,217/2,452	20–79	S	7
Colt, JS [21]	2011	US	2002–2007	CC	Any AHTN	RCC	1,201/2,427	20–79	S	6
Finkle, WD [56]	1993	US	1980–1989	CC	DU	RCC	191/382	59.6-case; 59.7-control (mean)	A	10
Flaherty, KT [57]	2005	US	1976–2000/ 1986–1998	Cohort	DU	RCC	265/167,144	42.4-NHS; 54-HPFS (mean)	A	6
Fraser, GE [58]	1990	US	1977–1982	Cohort	Any AHTN	RCC	14/34,198	72.3 (mean)	A	5
Friedman, GD [59]	2009	US	1994–2006	NCC	ACEI, CCB, DU	KC	572/630,311	NA	S	7
Fryzek, JP [60]	2005	Denmark	1989–2002	Cohort	ACEI, ARB, BB, CCB, DU, Any AHTN	RCC	330/113,298	NA	N	6
Hiatt, RA [61]	1994	US	1964–1989	CC	DU	RCC	257/514	50.7 (mean)	A	10
Hole, DJ [62]	1998	UK	1980–1995	Cohort	CCB	KC	15/5,207	51.7-M; 52.0-W (mean)	N	6
Jeon, HL [39]	2022	Korea	2002–2015	Cohort	ARB	KC	16,047/1,550,734	≥ 30	S	8
Jung, MH [40]	2021	Korea	2005–2012	Cohort	ARB	KC	744/293,962	≥ 40	S	10
Jung, M [63]	2024	US	2007–2021	Cohort	ACEI, ARB, CCB	KC	3,129/1,281,342 (1); 1,985/747,202 (2); 2,130/816,982 (3)^d^	58 (1); 59 (2); 59 (3)^d^ (median)	S	7
Kim, CS [40]	2020	Korea	2009–2017	Cohort	Any AHTN	KC	11,083/9,746,445	≥ 20	S	9
Kreiger, N [64]	1993	Canada	1986–1987	CC	DU	RCC	518/1,899	25–69	A	7
Kristensen, KB [11]	2020	Denmark	2000–2015	NCC	ACEI, ARB, BB, CCB, DU	RCC	7,315/153,615	18–85	A	8
Lindgren, AM [65]	2005	Finland	1972–1996	Cohort	Any AHTN	KC	66/20,529	51-M; 58-W (mean)	S	8
Mackenzie, IS [26]	2017	UK	1986–2013	Cohort	DU	RCC	136/222,225	NA	A	10
Matsui, S [20]	2021	Japan	2008–2015	Cohort	Any AHTN	KC	120/65,086	40–69	A	7
McCredie, M [27]	1992	Australia	1989–1990	CC	Any AHTN, BB, DU	RCC	636/1,159	20–79	A	7
McLaughlin, JK [66]	1995	Europe^b^, US	1989–1991	CC	DU, Non-DU	RCC	1,732/4,041	NA	A	9
Mellemgaard, A [67]	1994	Denmark	1989–1992	CC	ACEI, BB, CCB, DU, Any AHTN	RCC	368/764	NA	A, S	8
Nayan, M [13]	2017	Canada	1997–2014	NCC	ACEI, ARB, BB, CCB	KC	10,377/46,316	> 65	M, A	8
Prineas, RJ [25]	1997	US	1986–1993	Cohort	DU	RCC	62/35,192	55–69	S	7
Rosenberg, L [68]	1998	US	1976–1996	CC	ACEI, BB, CCB	KC	279/9,385	40–69	N	6
Schouten, LJ [22]	2005	Netherlands	1986–1997	Case-cohort	Any AHTN, BB, DU	RCC	337/4,774	NA	S	8
Setiawan, VW [69]	2007	US	1993–2002	Cohort	DU	RCC	347/161,126	45–75	A	8
Shapiro, JA [70]	1999	US	1980–1995	CC	ACEI, ARB, BB, CCB, DU, Non-DU	RCC	238/854	18–84	S	9
Weikert, S [45]	2008	Europe^c^	1992–1998	Cohort	Any AHTN	RCC	250/296,638	25–90	A	7
Weinmann, S [14]	1994	US	1960–1991	CC	BB, DU, Non-DU	RCC	206/498	36–85-M; 26–82-W	S	8
Yu, MC [24]	1986	US	1975–1979	CC	DU	RCC	160/320	15–54	A, S	6
Yuan, JM [71]	1998	US	1986–1994	CC	DU, Any AHTN, Non-DU	RCC	1,204/2,408	25–74	S	8
Zucchetto, A [72]	2007	Italy	1992–2004	CC	Any AHTN	RCC	767/2,301	62 (median)	N	2

Abbreviations: ACEI, angiotensin converting enzyme inhibitors; AHTN, antihypertensive medications; ARB, angiotensin receptor blockers; BB, beta-blockers; CC, case‒control study; CCB, calcium-channel blockers; DU, diuretics; HPFS, health professionals’ follow-up study; KC, kidney cancer; M, men; NHS, nurses’ health study; QS, quality score; RCC, renal cell carcinoma; W, women

^a^ Methods to account for hypertension: M, matching on hypertension; A, adjustment for hypertension; S, stratification/restriction to hypertensive status; N, no methods used to control hypertension

^b^ Australia, Denmark, Germany, Sweden

^c^ Denmark, France, Germany, Greece, Italy, Netherlands, Norway, Spain, Sweden, United Kingdom

^d^ [1] Cohort included ARB vs. ACEI; [2] Cohort included dihydropyridine CCB vs. ACEI; and [3] Cohort included dihydropyridine CCB vs. ARB

### Pooled effects of the overall analyses

The results from the overall analyses are shown in Table 2. We observed a significant pooled effect of an increased risk of KC for each class of AHTN (RR 1.29, 95% CI 1.04–1.62 for ACEI; RR 1.18, 95% CI 1.04–1.35 for ARB; RR 1.25, 95% CI 1.07–1.46 for BB, RR 1.44, 95% CI 1.20–1.72 for CCB; RR 1.42, 95% CI 1.31–1.54 for DU; and RR 1.50, 95% CI 1.22–1.86 for Any AHTN) compared to no use.

**Table 2:** Pooled associations between AHTN and kidney cancer by various methods used to account for hypertension

AHTN	Strata	No. of estimates^a^	No. of studies	Pooled RR (95% CI)	*P*	I^2^ value	*P* for difference^b^
ACEI	**Overall**	18	11	**1.29 (1.04–1.62)**	**0.03**	89.58	
	**None**	7	5	**1.66 (1.28–2.15)**	**0.03**	< 0.01	0.07
	**Any methods**	11	8	1.19 (0.93–1.52)	0.12	91.15	
	Matching/Adjustment for HTN	5	4	1.21 (0.93–1.57)	0.07	0.89	
	Stratified/Restricted to HTN population	5	4	1.23 (0.67–2.25)	0.30	93.74	
	Stratified/Restricted to non-HTN population	1	1	0.90 (0.29–2.80)	0.86	-	
ARB	**Overall**	10	8	**1.18 (1.04–1.35)**	**0.03**	85.50	
	**None**	2	2	**1.51 (1.45–1.58)**	**0.01**	< 0.01	**0.02**
	**Any methods**	8	7	**1.15 (1.00-1.31)**	**0.05**	74.02	
	Matching/Adjustment for HTN	2	2	**1.13 (1.02–1.25)**	**0.04**	< 0.01	
	Stratified/Restricted to HTN population	6	5	1.15 (0.87–1.51)	0.19	79.78	
	Stratified/Restricted to non-HTN population	0	0	-	-	-	
BB	**Overall**	22	13	**1.25 (1.07–1.46)**	**0.01**	71.05	
	**None**	10	8	**1.48 (1.24–1.77)**	**0.01**	7.69	**0.01**
	**Any methods**	12	9	**1.09 (1.03–1.16)**	**0.03**	0.01	
	Matching/Adjustment for HTN	7	6	1.10 (0.99–1.21)	0.05	0.01	
	Stratified/Restricted to HTN population	3	3	**1.09 (1.05–1.13)**	**0.01**	< 0.01	
	Stratified/Restricted to non-HTN population	2	2	0.97 (0.01–65.80)	0.94	62.12	
CCB	**Overall**	23	14	**1.44 (1.20–1.72)**	**< 0.01**	84.06	
	**None**	9	7	**1.75 (1.20–2.56)**	**0.03**	< 0.01	0.09
	**Any methods**	14	9	**1.40 (1.12–1.75)**	**0.01**	88.30	
	Matching/Adjustment for HTN	5	4	1.30 (0.93–1.81)	0.07	19.58	
	Stratified/Restricted to HTN population	8	5	1.45 (0.98–2.14)	0.06	89.59	
	Stratified/Restricted to non-HTN population	1	1	2.20 (0.90–5.39)	0.09	-	
DU	**Overall**	60	23	**1.42 (1.31–1.54)**	**< 0.01**	84.95	
	**None**	21	14	**1.54 (1.40–1.70)**	**< 0.01**	33.56	**0.03**
	**Any methods**	39	21	**1.36 (1.20–1.55)**	**< 0.01**	89.59	
	Matching/Adjustment for HTN	17	12	**1.38 (1.14–1.66)**	**< 0.01**	73.30	
	Stratified/Restricted to HTN population	17	10	**1.37 (1.04–1.80)**	**0.04**	94.92	
	Stratified/Restricted to non-HTN population	8	7	1.22 (0.83–1.81)	0.24	10.05	
Any AH TN	**Overall**	37	16	**1.50 (1.22–1.86)**	**< 0.01**	77.62	
	**None**	15	11	**1.64 (1.31–2.07)**	**< 0.01**	73.08	**0.04**
	**Any methods**	22	12	**1.40 (1.13–1.75)**	**0.01**	77.44	
	Matching/Adjustment for HTN	10	6	1.45 (0.86–2.42)	0.12	76.30	
	Stratified/Restricted to HTN population	7	6	1.44 (0.86–2.42)	0.13	73.74	
	Stratified/Restricted to non-HTN population	5	4	1.25 (0.69–2.24)	0.31	65.87	

Abbreviations: ACEI, angiotensin converting enzyme inhibitors; AHTN, antihypertensive medications; ARB, angiotensin receptor blockers; BB, beta-blockers; CCB, calcium-channel blockers; DU, diuretics; HTN, hypertension; RR, relative risk

^a^ To assess the independent effects of AHTN on KC risk from hypertension, we collected all available estimates based on the methods each contributing study used to account for hypertension: Any methods (matching, adjustment, or stratification/restriction) or none. For instance, we extracted both a crude estimate value and an adjusted value for hypertension from one study to compare with and without accounting for hypertension. To include all these values, we employed the robust variance estimate method with random effect models

^b^ Compared any methods versus none

### Pooled effects of the stratified analyses

**Fig. 1:**
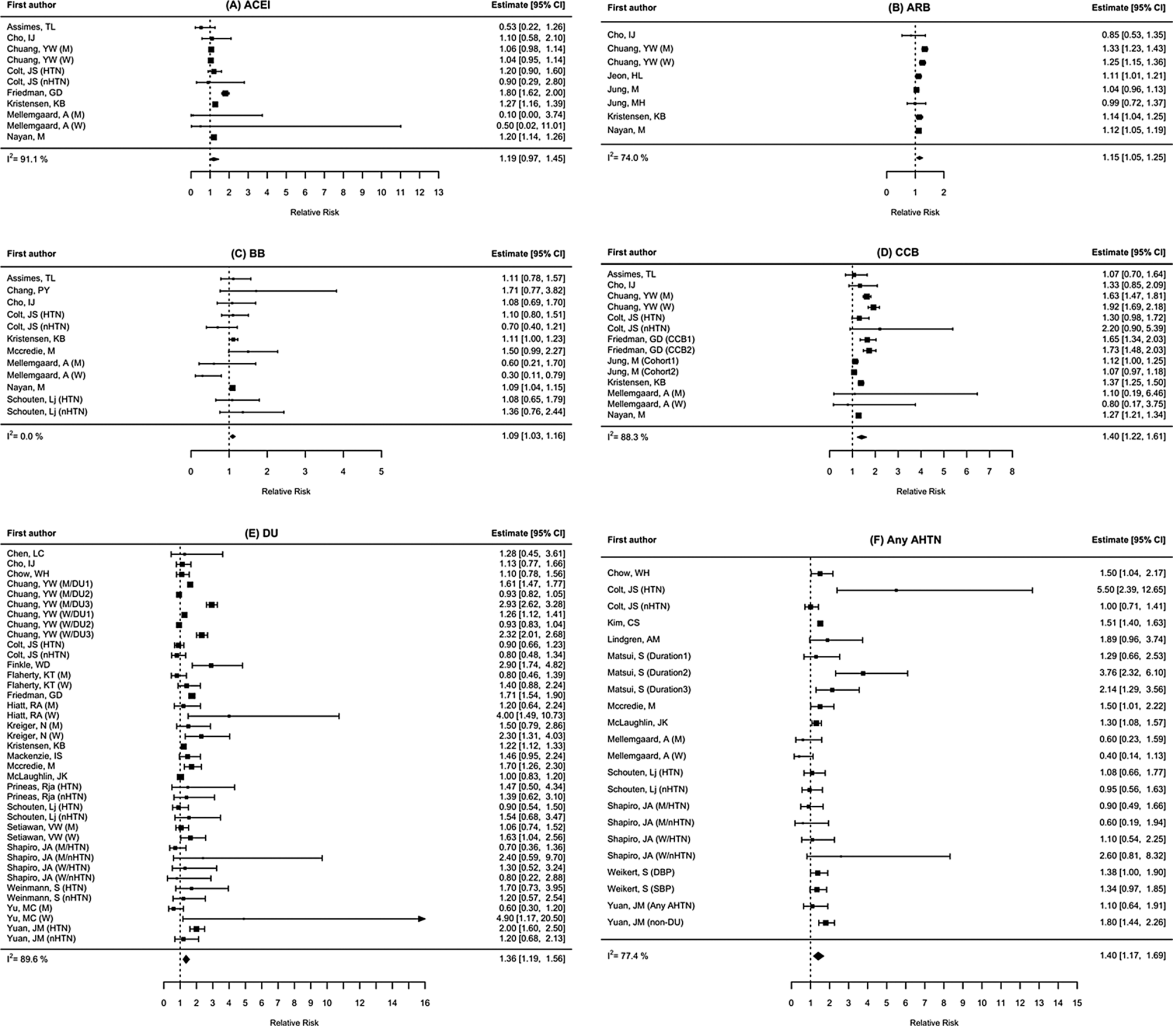
Forest plots of the associations between AHTN and kidney cancer accounting for hypertension. AHTN included (**A**) ACEI, (**B**) ARB, (**C**) BB, (**D**), CCB, (**E**) DU, and (**F**) Any AHTN. We found that significant pooled effects of ARB, BB, CCB, DU, and Any AHTN with an increased risk of KC, even after accounting for hypertension. Multiple estimates were chosen in several cases according to sex (men or women; M or W), stratification by hypertensive or nonhypertensive population (HTN or nHTN), different drugs of CCB or DU (CCB1-2 or DU1-3), different cohorts (Cohort1-2), duration of medication use (Duration1-3), or different adjustment for hypertension using systolic or diastolic blood pressure (SBP or DBP). Abbreviation. ACEI, angiotensin converting enzyme inhibitors; AHTN, antihypertensive medications; ARB, angiotensin receptor blockers; BB, beta-blockers; CCB, calcium-channel blockers; CI, confidence interval; DU, diuretics

Stratified analyses were conducted, presenting pooled estimates from studies that accounted for hypertension using any methods versus those that did not (Table 2). The pooled effects from estimates accounting for hypertension are shown in Fig. 1. Compared to no use, ARB (RR 1.15, 95% CI 1.00-1.31), BB (RR 1.09, 95% CI 1.03–1.16), CCB (RR 1.40, 95% CI 1.12–1.75), DU (RR 1.36, 95% CI 1.20–1.55), and Any AHTN (RR 1.40, 95% CI 1.13–1.75) were associated with an increased KC risk. Unlike other AHTN classes, ACEI did not show a statistically significant association with KC risk (RR 1.19, 95% CI 0.93–1.52).

In comparison, the pooled effects from estimates not accounting for hypertension showed stronger positive associations across all classes of AHTN (Table 2 and Figure S2). Compared to no use, ACEI (RR 1.66, 95% CI 1.28–2.15), ARB (RR 1.51, 95% CI 1.45–1.58), BB (RR 1.48, 95% CI 1.24–1.77), CCB (RR 1.75, 95% CI 1.20–1.26 1), DU (RR 1.54, 95% CI 1.40–1.70), and Any AHTN (RR 1.64, 95% CI 1.31–2.07) were associated with a higher KC risk.

The difference in pooled effects between the two strata from effect size estimates accounted for hypertension versus those that did not significant in ARB (*p* = 0.02), BB (*p* = 0.01), DU (*p* = 0.03), and Any AHTN (*p* = 0.04). Further stratified analyses by individual methods accounting for hypertension showed similar trends.

### Additional analyses

Similar pooled effects of AHTN use with an increased risk of KC were observed across predefined subgroups (Supplementary Materials, Table 5). In the analyses for ACEI, studies published before and after 2000 showed a significant difference (p for difference = 0.02), though both had null associations. In the analyses for ARB, the association of ARB use with KC risk was significantly different by sex (RR 1.25, 95% CI 1.15–1.36 for females and RR 1.33, 95% CI 1.23–1.43 for males; *p* = 0.02). Additionally, pooled estimates from studies with higher quality score showed a significant association of ARB with an increased risk of KC, while studies of lower quality showed no such association (*p* < 0.01). In the analyses for CCB, studies published after 2000 produced a significant positive pooled RR, while those published before 2000 showed a null RR (studies published after 2000 versus before 2000: RR 1.41, 95% CI 1.12–1.76 versus RR 0.92, 95% CI 0.29–2.94; *p* = 0.02).

After accounting for smoking or BMI in addition to hypertension, similar trends were shown across different classes of AHTN (Supplementary Materials, Table 6).

### Duration‒response analyses

The duration‒response analyses showed that AHTN use was associated with an increased the risk of KC by 2–6% with each additional year of AHTN use (ACEI: RR 1.04, 95% CI 1.04–1.04; ARB: RR 1.06, 95% CI 1.05–1.06; BB: RR 1.02, 95% CI 1.01–1.02; and CCB: RR 1.04, 95% CI 1.04–1.04), while DU and Any AHTN showed no significant association (Table 3 and Figure S3). Similar results were shown after accounting for hypertension.

**Table 3:** Duration-response relationships between one-year increments in AHTN exposure and kidney cancer risk

	No. of Estimates	No. of studies	Pooled RR (95% CI) including all estimates	*P*	Pooled RR (95% CI) based on estimates thataccounted for hypertension	*P*
ACEI	13	2	**1.04 (1.04–1.04)**	**< 0.01**	**1.04 (1.04–1.04)**	**< 0.01**
ARB	15	2	**1.06 (1.05–1.06)**	**< 0.01**	**1.06 (1.05–1.06)**	**< 0.01**
BB	17	3	1.02 (1.00-1.04)	0.05	**1.02 (1.01–1.02)**	**< 0.01**
CCB	23	3	**1.04 (1.04–1.04)**	**< 0.01**	**1.04 (1.04–1.04)**	**< 0.01**
DU	43	8	1.01 (0.97–1.05)	0.48	1.01 (0.97–1.06)	0.33
Any AHTN	14	4	1.01 (0.96–1.07)	0.26	1.02 (0.92–1.12)	0.41

Abbreviation. ACEI, angiotensin converting enzyme inhibitors; AHTN, antihypertensive medications; ARB, angiotensin receptor blockers; BB, beta-blockers; CCB, calcium-channel blockers; DU, diuretics; RR, relative risk

### Publication bias assessment

**Fig. 2:**
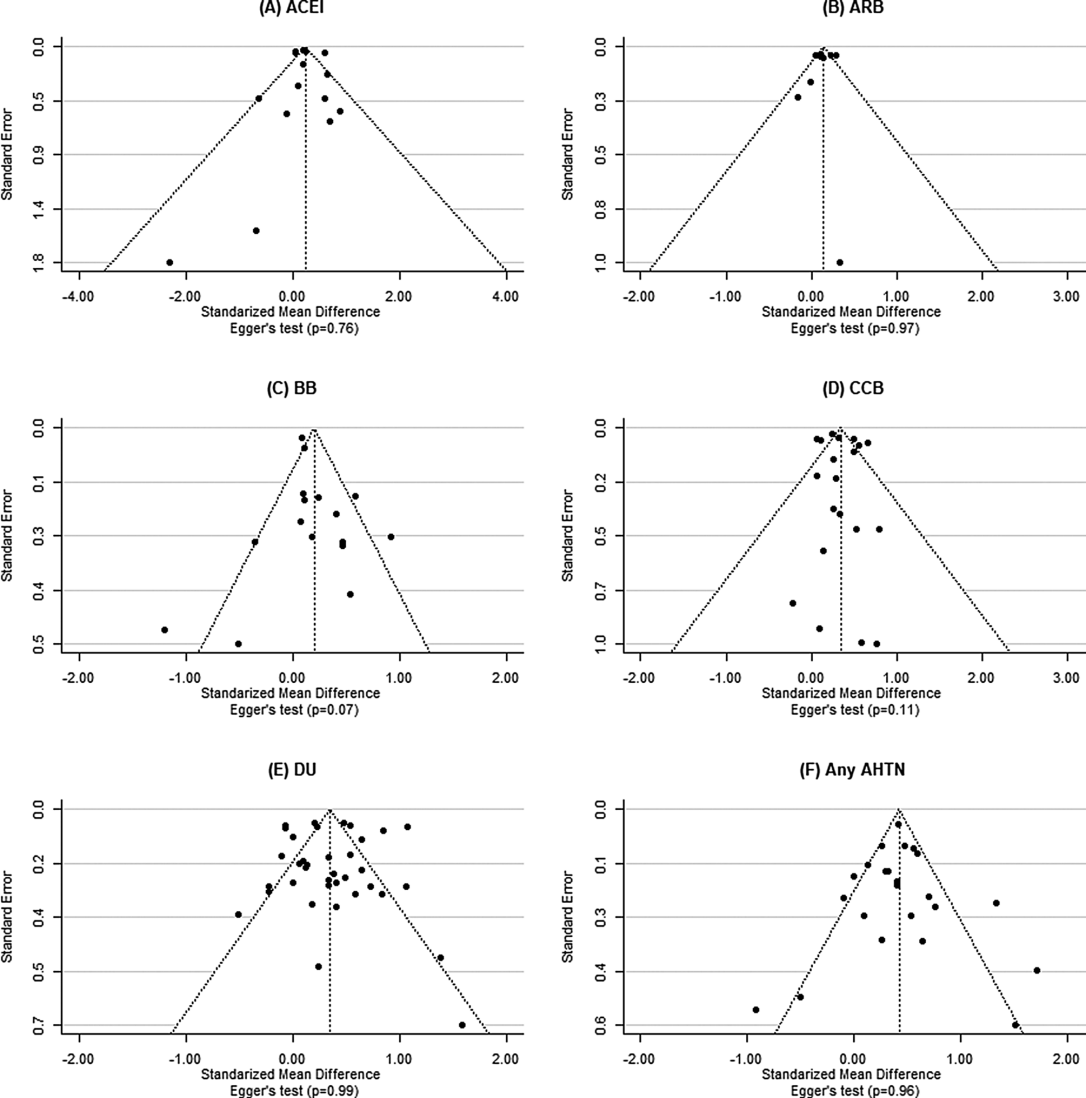
Begg’s funnel plots with Egger’s regression tests to assess potential publication bias. For each AHTN (**A**-**F**), publication bias is unlikely in our meta-analysis. Abbreviation. ACEI, angiotensin converting enzyme inhibitors; ARB, angiotensin receptor blocker; BB, beta-blocker; CCB, calcium-channel blocker; DU, diuretic

Begg’s funnel plots showed a symmetric distribution, and Egger’s test was not statistically significant for each AHTN, implying that publication bias is unlikely in our meta-analysis (Fig. 2).

### Sensitivity analysis

Two sensitivity analyses for potential overlapping population issues (Table S7) and leave-one-out sensitivity analysis (Table S8) confirmed robustness.

## Discussion

Our meta-analysis found that AHTN use was associated with a higher risk of KC, even after accounting for hypertension, with the highest risk for CCB and the lowest for ACEI. By differentiating results based on whether hypertension was accounted for through matching, stratification/restriction, or statistical adjustment, our study ensures the associations between AHTN use and an increased KC risk are not solely driven by high blood pressure but may represent independent associations. Results when further accounting for smoking and BMI and those from duration‒response analysis and sensitivity analyses confirmed robustness.

Previous studies have supported our findings. In a previous meta-analysis, the use of ARB (RR 1.29, 95% CI 1.22–1.37), CCB (RR 1.70, 95% CI 1.57–1.83), and DU (RR 1.35, 95% CI 1.17–1.54) were associated with an increased risk of KC [15]. In another meta-analysis, a similar result between DU and KC risk was observed (RR 1.34, 95% CI 1.13–1.58) [43]. However, they have several limitations. First, despite the availability of various methods to control confounders [44], they addressed hypertension only through adjustment method and subgroup analyses, not in the main analysis. Their main findings might still be confounded by hypertension. Second, they disregarded potential dependencies: (1) multiple estimates from a single study, such as several non-exclusive exposures assessed within the same population, shared comparison groups, or repeated measures over time; and (2) dependencies between studies using the same database, potentially sharing the same population. Ignoring these dependencies can distort standard errors, inflating Type 1 error rates and misrepresenting confidence intervals [34, 35]. 

In contrast, we addressed potential confounding effects of hypertension using various methods, including matching and stratification/restriction in addition to adjustment. In addition, we applied the random-effects models with RVE to account for the intra- and inter-study dependencies. Our study is the most comprehensive to date, incorporating recent research, particularly highlighting novel findings that present pooled results based on estimates with and without accounting for hypertension. AHTN use was significantly associated with a 48–75% increased risk of KC when not accounting for hypertension, which attenuated to 9–40% but remained significant when hypertension was accounted for. The increased risks indicate that the KC risk associated with AHTN use may be independent of hypertension. Notably, this attenuation suggests that hypertension itself still plays a key role in KC risk, and residual confounding cannot be ruled out. Among different classes of AHTN, CCB showed the strongest association with KC risk, while ACEI showed the lowest (null association). Additionally, duration-response analyses showed a significant 2–6% increase in KC risk for each additional year of prolonged AHTN use, except for DU and Any AHTN. These findings emphasize the need for close monitoring of patients on lifelong therapy and careful risk-benefit assessments.

Significant differences were observed by publication year for ACEI and CCB, and by sex and quality score for ARB, suggesting potential sources of heterogeneity in our meta-analysis. Higher quality and recently published studies strengthened the robustness of our results. In male ARB users, the association with an increased KC risk was stronger than in females, though both showed elevated risks. Pooled estimates remained consistent across outcome types, drug exposure assessment methods, study designs, estimate types, or geographical regions, confirming the validity of the main results. Subgroup analyses for obesity and smoking history were not feasible due to limited data, but stratified analyses with adjusting for these factors confirmed robustness.

Despite this potential harm, adherence to AHTN treatment remains clinically important, as the impact of hypertension on the risk of KC has been known to be greater than that attributed to AHTN use [6, 12, 45]. High blood pressure increased KC risk by approximately 2.5-fold compared to low blood pressure [45] and showed a dose-dependent relationship for 8 years of follow-up [12]. Our findings, showing the attenuation of risk estimates after accounting for hypertension, also support this. Thus, it is important to ensure that optimal blood pressure control remains the primary focus, with an awareness of any potential risks associated with long-term AHTN use.

Our findings indicate that KC risks vary across different AHTN classes. This suggests that selecting the appropriate AHTN is crucial and could be a modifiable factor. The cautious use of CCB, particularly for long-term treatment, is recommended due to associated risks. For clinicians managing high-risk patients, such as those with hypertension, obesity, or a smoking history, ACEI may be a preferable treatment option unless specific contraindications exist. ACEI has shown minimal to no association with increased KC risk, suggesting it is potentially a safer option for these patients. Understanding patients’ absolute risks of KC with and without AHTN use, as well as their cardiovascular disease risk is crucial to balance the benefits and potential harms of AHTN therapy. Further clinical trials, prospective studies, and the development of risk prediction models are needed to guide personalized AHTN therapy, ensuring cardiovascular benefits while addressing KC concerns.

Although the carcinogenic pathogenesis of AHTN in KC development has not yet been established, several plausible mechanisms have been proposed. As each class of AHTN displays distinct mechanisms of action, each may affect KC risk differently. To date, DU shows strong evidence for kidney tumorigenesis, potentially converting to a mutagenic nitroso derivative in the stomach [18], acting as a carcinogen in renal tubular cells [46], and causing cellular hypertrophy of the proximal tubules after potassium depletion [47]. Studies on rodents and renal biopsies from exposed patients show evidence of developing renal adenomas and nephropathy [48], and renal interstitial fibrosis and inflammation [49]. CCB may affect cancer cell differentiation by modulating intracellular calcium levels, which regulates various cellular processes, including signal transduction, gene expression, and cell cycle progression [17, 50]. Alteration of calcium levels may inhibit apoptosis, allowing damaged cells to survive longer than they would in a normal, healthy system, and increasing tumorigenesis risk [17, 51]. ARB may promote carcinogenesis through upregulation of angiotensin II type 2 receptor (AT2R) when AT1R is blocked, leading to cellular proliferation, migration, and fibrosis [16]. ACEI may facilitate KC development by increasing bradykinin levels, which are associated with inflammation and immune response, potentially influencing tumor progression [52]. In contrast, ACEI and ARB may also have anticancer effects by downregulating vascular endothelial growth factor-mediated angiogenesis; ACEI reduces angiotensin II levels, and ARB blocks AT1R. This leads to inhibition of cellular proliferation, migration, and angiogenesis, potentially limiting tumor growth and progression [53]. The balance between these mechanisms of ACEI and ARB related to KC is complex, multifaceted, and not yet fully understood. There is limited evidence on the role of BB in renal carcinogenesis.

We have several limitations. First, although subgroup analyses suggested potential sources of heterogeneity (e.g., sex, publication year) but did not fully explain it. This is possibly due to differences in study designs, methods, study populations (e.g., diverse races/ethnicities), and healthcare practices and systems. Variations may also result from how several confounders (e.g., compelling indications for AHTN use, hypertension severity, genetic predispositions, or lifestyle factors) were handled. Future prospective studies or Mendelian randomization studies addressing these confounders are needed. Second, limited data prevented analysis by diuretic subtypes (e.g., thiazide, loop, and potassium-sparing) [9, 48], hypertension duration, different AHTN treatment regimens (dosage, multitherapy), which may indicate severe hypertension or other underlying conditions and could provide more detailed outcomes. Third, since our findings are from observational studies, they are subject to limitations such as residual confounding, selection bias, reverse causation, and the inability to establish causality. In addition, one study was excluded for being written in a non-English language, and we believe language bias will be minimal.

## Conclusions

This meta-analysis, including 39 observational studies, found that AHTN use was associated with an increased risk of KC, even after accounting for hypertension, though residual confounding cannot be ruled out. Among AHTN classes, CCB showed the highest increased risk of KC, while ACEI use showed no significant association. These findings were further supported by duration-response relationships. Our findings highlight the potential KC risks associated with AHTN use, with optimal cardiovascular care remaining an important consideration.

## Electronic supplementary material

Below is the link to the electronic supplementary material.

Supplementary Material 1

## Data Availability

The information of datasets used for the current meta-analysis are available from the corresponding author upon reasonable request.

## Abbreviations

AHTN: Antihypertensive medication
ACEI: Angiotensin-converting enzyme inhibitor
ARB: Angiotensin receptor blocker
AT1R: Angiotensin II type 1 receptor
AT2R: Angiotensin II type 2 receptor
BB: Beta-blocker
BMI: Body mass index
CCB: Calcium channel blocker
CI: Confidence interval
DU: Diuretic
KC: Kidney cancer
OR: Odds ratio
RCC: Renal cell carcinoma
RR: Relative risk
RVE: Robust variance estimation
