# Supplementary material for: Association between antihypertensive medication use and kidney cancer risk: a meta-analysis accounting for hypertension
Source: BMC Cancer. 2025 Jun 6;25:1013. doi: 10.1186/s12885-025-14406-3 (PMC12143101; doi:10.1186/s12885-025-14406-3)
Supplement: Supplementary file 1 — Supplementary Material 1 [file 12885_2025_14406_MOESM1_ESM.docx]

**Supplemental Materials**

**Supplementary Table 1.** PRISMA (Preferred Reporting Items for Systematic Reviews and Meta-Analyses) Checklist

**Supplementary Table 2**. Literature search strategies using PubMed, Embase, Web of Science, and Cochrane

**Supplementary Table 3**. Scores of quality assessment based on the revised version of Newcastle-Ottawa Quality Assessment scale

**Supplementary Table 4**. Summary table of characteristics of included studies according to the class of antihypertensive medication

**Supplementary Table 5**. Pooled associations between AHTN and kidney cancer by pre-defined subgroups using all available estimates

**Supplementary Table 6.** Pooled estimates stratified by adjustment for smoking or body mass index in addition to hypertension

**Supplementary Table 7**. Sensitivity analyses for potential overlapping population issues

**Supplementary Table 8**. Leave-one-out sensitivity analysis for potential issues with highly influential studies

**Supplementary Figure 1**. Flowchart of article screening and selection process along with inclusion and exclusion criteria

**Supplementary Figure 2**. Forest plots of pooled estimates using all available estimates for the stratified analyses without accounting for hypertension

**Supplementary Figure 3**. Bubble plots for dose-response relationships between antihypertensive medication use and kidney cancer risk

**Supplementary Table 1**. PRISMA (Preferred Reporting Items for Systematic Reviews and Meta-Analyses) Checklist

| **Section/Topic** | **Item No.** | **Checklist item** | **Reported on Page No.** | |
| --- | --- | --- | --- | --- |
| **TITLE** | | | | |
| Title | 1 | Identify the report as a systematic review, meta-analysis, or both. | 1 | |
| **ABSTRACT** | | | | |
| Structured summary | 2 | Provide a structured summary including, as applicable: background; objectives; data sources; study eligibility criteria, participants, and interventions; study appraisal and synthesis methods; results; limitations; conclusions and implications of key findings; systematic review registration number. |  | |
|  |  |  | 2 | |
|  |  |  |  | |
| **INTRODUCTION** | | | | |
| Rationale | 3 | Describe the rationale for the review in the context of what is already known. | 5 | |
| Objectives | 4 | Provide an explicit statement of questions being addressed with reference to participants, interventions, comparisons, outcomes, and study design (PICOS). |  | |
|  |  |  | 5 | |
|  |  |  |  | |
| **METHODS** | | | | |
| Protocol and registration | 5 | Indicate if a review protocol exists, if and where it can be accessed (e.g., Web address), and, if available, provide registration information including registration number. |  | |
|  |  |  | N/A | |
|  |  |  |  | |
| Eligibility criteria | 6 | Specify study characteristics (e.g., PICOS, length of follow-up) and report characteristics (e.g., years considered, language, publication status) used as criteria for eligibility, giving rationale. |  | |
|  |  |  | 6 | |
|  |  |  |  | |
| Information sources | 7 | Describe all information sources (e.g., databases with dates of coverage, contact with study authors to identify additional studies) in the search and date last searched. |  | |
|  |  |  | 6 | |
|  |  |  |  | |
| Search | 8 | Present full electronic search strategy for at least one database, including any limits used, such that it could be repeated. |  | |
|  |  |  | 6 | |
|  |  |  |  | |
| Study selection | 9 | State the process for selecting studies (i.e., screening, eligibility, included in systematic review, and, if applicable, included in the meta-analysis). |  | |
|  |  |  | 6,7 | |
|  |  |  |  | |
| Data collection process | 10 | Describe method of data extraction from reports (e.g., piloted forms, independently, in duplicate) and any processes for obtaining and confirming data from investigators. |  | |
|  |  |  | 7,8 | |
|  |  |  |  | |
| Data items | 11 | List and define all variables for which data were sought (e.g., PICOS, funding sources) and any assumptions and simplifications made. |  | |
|  |  |  | 7,8 | |
| Risk of bias in individual studies | 12 | Describe methods used for assessing risk of bias of individual studies (including specification of whether this was done at the study or outcome level), and how this information is to be used in any data synthesis. |  | |
|  |  |  | 8,9 | |
|  |  |  |  | |
|  |  |  |  | |
| Summary measures | 13 | State the principal summary measures (e.g., risk ratio, difference in means). | 8 | |
| Synthesis of results | 14 | Describe the methods of handling data and combining results of studies, if done, including measures of consistency (e.g., I2) for each meta-analysis. | 9 | |
| Risk of bias across studies | 15 | Specify any assessment of risk of bias that may affect the cumulative evidence (e.g., publication bias, selective reporting within studies). | 9 | |
| Additional analyses | 16 | Describe methods of additional analyses (e.g., sensitivity or subgroup analyses, meta-regression), if done, indicating which were pre-specified. | 8,9 | |
| **RESULTS** | | | | |
| Study selection | 17 | Give numbers of studies screened, assessed for eligibility, and included in the review, with reasons for exclusions at each stage, ideally with a flow diagram. |  | |
|  |  |  | 10 | |
|  |  |  |  | |
| Study characteristics | 18 | For each study, present characteristics for which data were extracted (e.g., study size, PICOS, follow-up period) and provide the citations. |  | |
|  |  |  | 10 | |
|  |  |  |  | |
| Risk of bias within studies | 19 | Present data on risk of bias of each study and, if available, any outcome level assessment (see item 12). |  | |
|  |  |  | 12 | |
|  |  |  |  | |
| Results of individual studies | 20 | For all outcomes considered (benefits or harms), present, for each study: (a) simple summary data for each intervention group  (b) effect estimates and confidence intervals, ideally with a forest plot. |  | |
|  |  |  | F1 | |
|  |  |  |  | |
| Synthesis of results | 21 | Present results of each meta-analysis done, including confidence intervals and measures of consistency. | 10,11 | |
|  |  |  |  | |
| Risk of bias across studies | 22 | Present results of any assessment of risk of bias across studies (see Item 15). |  | |
|  |  |  | 12 | |
|  |  |  |  | |
| Additional analysis | 23 | Give results of additional analyses, if done (e.g., sensitivity or subgroup analyses, meta-regression [see Item 16]). |  | |
|  |  |  | 11,12 | |
|  |  |  |  | |
| **DISCUSSION** | | | | |
| Summary of evidence | 24 | Summarize the main findings including the strength of evidence for each main outcome; consider their relevance to key groups (e.g., healthcare providers, users, and policy makers). |  | |
|  |  |  | 13-16 | |
|  |  |  |  | |
| Limitations | 25 | Discuss limitations at study and outcome level (e.g., risk of bias), and at review-level (e.g., incomplete retrieval of identified research, reporting bias). |  | |
|  |  |  | 16,17 | |
|  |  |  |  | |
| Conclusions | 26 | Provide a general interpretation of the results in the context of other evidence, and implications for future research. |  | |
|  |  |  | 17 | |
|  |  |  |  | |
| **FUNDING** | | | | |
| Funding | 27 | Describe sources of funding for the systematic review and other support (e.g., supply of data); role of funders for the systematic review. | |  |
|  |  |  |  | 4 |
|  |  |  |  |  |

*From:* Moher D, Liberati A, Tetzlaff J, Altman DG, The PRISMA Group (2009). Preferred Reporting Items for Systematic Reviews and Meta-Analyses: The PRISMA Statement. PLoS Med 6(6): e1000097. doi:10.1371/journal.pmed1000097

**Supplementary Table 2**. Literature search strategies using PubMed, Embase, Web of Science, and Cochrane

| **PubMed** (N=3,224 studies) |
| --- |
| ("Adrenergic beta-Antagonists"[Mesh] OR "Angiotensin-Converting Enzyme Inhibitors"[Mesh] OR "Diuretics"[Mesh] OR "Antihypertensive Agents"[Mesh] OR "Antihypertensive Agents"[tw] OR antihypertensive[tw] OR "anti-hypertensive"[tw] OR antihypertensives[tw] OR “antihypertensive drug*”[tw] OR “antihypertensive medication*”[tw] OR “anti-hypertensives”[tw] OR “anti-hypertensive drug*”[tw] OR “anti-hypertensive medication*”[tw] OR "Angiotensin II Type 1 Receptor Blockers"[mesh] OR "Calcium Channel Blocker*"[tw] OR "calcium channel blocker*"[mesh] OR "angiotensin II type 1 receptor blocker*"[tw] OR "Adrenergic beta-Antagonist*"[tw] OR "Angiotensin-Converting Enzyme Inhibitor*"[tw] OR "Diuretic*"[tw] OR “renin angiotensin aldosterone system inhibitor*”[tw] OR “renin-angiotensin-aldosterone system inhibitor*”[tw] OR “ACEI*”[tw] OR “ACE inhibitor*”[tw] OR “angiotensin II receptor blocker*”[tw] OR “ARB*”[tw] OR “BB”[tw] OR “BBs”[tw] OR “beta-blocker*”[tw] OR “beta blocker*”[tw] OR “CCB*”[tw] OR “calcium-channel blocker*”[tw] OR “thiazide*”[tw] OR “spironolactone”[tw]) AND (("Kidney Neoplasms"[mesh] OR "renal cancer*"[tw] OR "renal tumor*" OR "renal tumour" OR "kidney tumor" OR "kidney tumour" OR "kidneys tumor" OR "urologic neoplasms"[mesh] OR “Renal Cell Carcinoma*” OR “RCC”) OR ((renal[tw] OR “renal cell"[tw] OR kidney*[tw] OR “urinary tract” [tw]) AND (neoplasm*[tw] OR cancer*[tw] OR tumor[tw] OR malignanc*[tw] OR tumour[tw] OR carcinoma[tw]))) NOT (“review”[pt] OR “editorial”[pt] OR “guideline”[pt]) NOT (“animals”[mesh] NOT “humans”[mesh]) |
| **Embase** (N=8,757 studies) |
| ('antihypertensive agent'/exp OR 'dipeptidyl carboxypeptidase inhibitor'/exp OR antihypertensive*:ti,ab,kw OR 'anti-hypertensive*':ti,ab,kw OR 'antihypertensive medication*':ti,ab,kw OR 'angiotensin receptor antagonist'/exp OR 'calcium channel blocker*':ti,ab,kw OR 'calcium channel blocking agent'/exp OR 'angiotensin 2 receptor antagonist'/exp OR 'angiotensin 2 receptor antagonist*':ti,ab,kw OR 'angiotensin ii type 1 receptor blocker*':ti,ab,kw OR 'adrenergic beta-antagonist*':ti,ab,kw OR 'angiotensin-converting enzyme inhibitor*':ti,ab,kw OR 'diuretic agent'/exp OR 'diuretic*':ti,ab,kw OR 'renin angiotensin aldosterone system inhibitor*':ti,ab,kw OR 'renin-angiotensin-aldosterone system inhibitor*':ti,ab,kw OR 'acei*':ti,ab,kw OR 'ace inhibitor*':ti,ab,kw OR 'angiotensin ii receptor blocker*':ti,ab,kw OR 'arb*':ti,ab,kw OR 'beta adrenergic receptor blocking agent'/exp OR 'beta-blocker*':ti,ab,kw OR 'beta blocker*':ti,ab,kw OR 'ccb*':ti,ab,kw OR 'calcium-channel blocker*':ti,ab,kw OR 'thiazide*':ti,ab,kw OR 'spironolactone':ti,ab,kw) AND ('kidney cancer'/exp OR 'urinary tract cancer'/de OR 'rcc':ti,ab,kw OR ((renal:ti,ab,kw OR 'renal cell':ti,ab,kw OR kidney*:ti,ab,kw OR nephro*:ti,ab,kw OR 'urinary tract':ti,ab,kw) AND (neoplasm*:ti,ab,kw OR cancer*:ti,ab,kw OR tumor:ti,ab,kw OR malignanc*:ti,ab,kw OR tumour:ti,ab,kw OR carcinoma:ti,ab,kw OR neoplas*:ti,ab,kw))) NOT ('animal'/exp NOT 'human'/exp) AND ([article]/lim OR [article in press]/lim OR [data papers]/lim) |
| **Web of Science** (N=3,000 studies) |
| (TS=((antihypertensive* OR “anti-hypertensive*” OR ((adrenergic OR angiotensin OR “angiotensin II” OR “angiotensin 2”) NEAR/3 (antagonist* OR inhibitor* OR blocker*)) OR “dipeptidyl carboxypeptidase inhibitor*” OR “calcium channel block*” OR diuretic* OR “renin angiotensin aldosterone system inhibitor*” OR “renin-angiotensin-aldosterone system inhibitor*” OR “acei*” OR “ace inhibitor*” OR “arb*” OR “beta-blocker*” OR “beta blocker*” OR “ccb*” OR “calcium-channel blocker*” OR “thiazide*” OR “spironolactone”) AND (“rcc” OR ((renal OR kidney* OR “renal cell” OR nephro* OR “urinary tract”) AND (cancer* OR tumor OR tumour OR carcinoma* OR lymphoma OR carcinoma OR malignan* OR neoplasm*))) )) AND (DT==("ARTICLE" OR "EARLY ACCESS" OR "CORRECTION")) |
| **Cochrane** (N=1,474 studies) |
| (antihypertensive* OR “anti-hypertensive*” OR ((adrenergic OR angiotensin OR “angiotensin II” OR “angiotensin 2”) NEAR/3 (antagonist* OR inhibitor* OR blocker*)) OR “dipeptidyl carboxypeptidase inhibitor*” OR “calcium channel block*” OR diuretic* OR “renin angiotensin aldosterone system inhibitor*” OR “renin-angiotensin-aldosterone system inhibitor*” OR “acei*” OR “ace inhibitor*” OR “arb*” OR “beta-blocker*” OR “beta blocker*” OR “ccb*” OR “calcium-channel blocker*” OR “thiazide*” OR “spironolactone”) AND (“rcc” OR ((renal OR kidney* OR “renal cell” OR nephro* OR “urinary tract”) AND (cancer* OR tumor OR tumour OR carcinoma* OR lymphoma OR carcinoma OR malignan* OR neoplasm*))) |

**Supplementary Table 3.** Scores of quality assessment based on the revised version of Newcastle-Ottawa Quality Assessment scale

|  | **Population and Case/Outcome** | | | **Confounder** | | **Risk factor/Exposure and Statistics** | | | |  |
| --- | --- | --- | --- | --- | --- | --- | --- | --- | --- | --- |
| **First Author (year)** | **Representativeness of population** | **Selection of study groups from the same population** | **Ascertainment of kidney cancer incidence^a^** | **Methods of accounting for hypertension, smoking, and BMI^b^** | **Methods of accounting for age and sex^c^** | **Adequacy of analyzing individual AHTN^d^** | **Ascertainment of AHTN^e^** | **Ascertainment of comparator^e^** | **Adequacy of reporting statistical results (confidence interval or standard error)** | **Total Sum** |
| Assimes (2008) | 1 | 1 | 1 | 1 | 1 | 1 | 1 | 1 | 0 | 8 |
| Braun (1998) | 0 | 1 | 1 | -1 | 0 | 1 | 1 | 1 | -1 | 3 |
| Chang (2015) | 1 | 1 | 1 | 1 | 1 | 1 | 1 | 1 | 0 | 8 |
| Chen (2024) | 1 | 1 | 1 | 1 | 1 | 1 | 1 | 1 | 0 | 8 |
| Cho (2021) | 1 | 1 | 1 | 3 | 1 | 1 | 1 | 1 | 0 | 10 |
| Chow (1995) | 1 | 0 | 1 | 3 | 1 | 1 | 0 | 0 | 0 | 7 |
| Chuang (2017) | 1 | 1 | 1 | 1 | 1 | 1 | 1 | 1 | 0 | 8 |
| Colt (2011) | 1 | 0 | 1 | 3 | 1 | 0 | 1 | 1 | 0 | 8 |
| Colt (2017) | 1 | 0 | 1 | 3 | 1 | 1 | 1 | 1 | 0 | 9 |
| Finkle (1993) | 1 | 1 | 1 | 3 | 1 | 1 | 1 | 1 | 0 | 10 |
| Flaherty (2005) | 0 | 1 | 1 | 2 | 1 | 1 | 0 | 0 | 0 | 6 |
| Fraser (1990) | 1 | 1 | 1 | -1 | 1 | 0 | 0 | 0 | 0 | 3 |
| Friedman (2009) | 0 | 1 | 1 | -1 | 1 | 1 | 1 | 1 | 0 | 5 |
| Fryzek (2005) | 1 | 1 | 1 | -1 | 1 | 1 | 1 | 1 | 0 | 6 |
| Hiatt (1994) | 1 | 1 | 1 | 3 | 1 | 1 | 1 | 1 | 0 | 10 |
| Hole (1998) | 1 | 0 | 1 | -1 | 1 | 1 | 1 | 1 | 0 | 5 |
| Jeon (2022) | 1 | 1 | 1 | 1 | 1 | 1 | 1 | 1 | 0 | 8 |
| Jung (2020) | 1 | 1 | 1 | 3 | 1 | 1 | 1 | 1 | 0 | 10 |
| Jung (2024) | 0 | 1 | 1 | 1 | 1 | 1 | 1 | 1 | 0 | 7 |
| Kim (2020) | 1 | 1 | 1 | 3 | 1 | 0 | 1 | 1 | 0 | 9 |
| Kreiger (1993) | 1 | 0 | 1 | 3 | 1 | 1 | 0 | 1 | 0 | 8 |
| Kristensen (2020) | 1 | 1 | 1 | 1 | 1 | 1 | 1 | 1 | 0 | 8 |
| Lindgren (2005) | 1 | 1 | 1 | 2 | 1 | 0 | 1 | 1 | 0 | 8 |
| Mackenzie (2017) | 1 | 1 | 1 | 3 | 1 | 1 | 1 | 1 | 0 | 10 |
| Matsui (2021) | 1 | 1 | 1 | 3 | 1 | 0 | 0 | 0 | 0 | 7 |
| McCredie (1992) | 1 | 0 | 1 | 3 | 1 | 1 | 1 | 1 | 0 | 9 |
| McLaughilin (1995) | 1 | 0 | 1 | 3 | 1 | 1 | 1 | 1 | 0 | 9 |
| Mellemgaard (1994) | 1 | 0 | 1 | 3 | 1 | 1 | 0 | 0 | 0 | 7 |
| Nayan (2017) | 1 | 1 | 1 | -1 | 1 | 1 | 1 | 1 | 0 | 6 |
| Prineas (1997) | 1 | 1 | 1 | 2 | 1 | 1 | 0 | 0 | 0 | 7 |
| Rosenberg (1998) | 1 | 1 | 1 | -1 | 1 | 1 | 1 | 1 | 0 | 6 |
| Schouten (2005) | 1 | 1 | 1 | -1 | 1 | 1 | 0 | 0 | 0 | 4 |
| Setiawan (2007) | 1 | 1 | 1 | 3 | 1 | 1 | 0 | 0 | 0 | 8 |
| Shapiro (1999) | 1 | 1 | 1 | 3 | 1 | 1 | 1 | 1 | 0 | 10 |
| Weikert (2007) | 1 | 1 | 1 | 3 | 1 | 0 | 0 | 0 | 0 | 7 |
| Weinmann (1994) | 1 | 1 | 1 | 1 | 1 | 1 | 1 | 1 | 0 | 8 |
| Yu (1986) | 1 | 0 | 1 | 2 | 1 | 1 | 0 | 0 | 0 | 6 |
| Yuan (1998) | 1 | 0 | 1 | 2 | 1 | 1 | 1 | 1 | 0 | 8 |
| Zucchetto (2007) | 0 | 1 | 1 | -1 | 1 | 0 | 0 | 0 | 0 | 2 |
| **Note**. We assessed study quality using a revised Newcastle-Ottawa scale based on three criteria: 1) clinical or histological confirmation of KC, 2) control for confounders (hypertension, smoking, BMI, age, sex), and 3) identification of individual AHTN classes. A score of ≤7 was considered low to moderate quality, and 8-10 was high quality.  **Abbreviations**. AHTN, antihypertensive medications; BMI, body mass index  **^a^** Whether incidence of kidney cancer was ascertained by diagnosis record and histological confirmation (1).  **^b^** If hypertension, smoking, and body mass index (BMI) were accounted, then (3); if two of them (hypertension and either one of smoking or BMI) were accounted, then (2); if only hypertension was considered, then (1); or if hypertension was not accounted, then (-1).  **^c^** If age and sex were matched or adjusted, then (1).  **^d^** If a study presented results of each AHTN separately, then (1).  **^e^** Whether antihypertensive medication use or no use was ascertained by medical records reported (1) and self-reported/interviewed (0). | | | | | | | | | | |

**Supplementary Table 4.** Summary table of characteristics of studies included according to the class of antihypertensive medication

| **Class of antihypertensives** | **Number of studies included** | **KC cases/ participants** | **Study design ^a^ (Cohort/ Case-control)** | **Cancer type (RCC/KC)** | **Geographical region ^b^**  **(US or Canada/**  **Others)** | **Published year (Before/**  **After 2000)** | **Quality score**  **(Low-moderate/ High)** |
| --- | --- | --- | --- | --- | --- | --- | --- |
| **ACEI** | 11 | 27,643/241,509 | 7/4 | 5/6 | 6/5 | 3/8 | 4/7 |
| **ARB** | 8 | 31,506/2,172,013 | 8/0 | 2/6 | 2/6 | 0/8 | 2/6 |
| **BB** | 13 | 19,162/229,755 | 6/7 | 8/5 | 6/7 | 5/8 | 4/9 |
| **CCB** | 14 | 30,002/1,121,731 | 10/4 | 5/9 | 7/7 | 5/9 | 7/7 |
| **DU** | 23 | 57,482/643,967 | 10/13 | 20/3 | 13/10 | 13/10 | 9/14 |
| **Any AHTN** | 16 | 14,616/6,024,135 | 6/10 | 13/3 | 6/10 | 9/7 | 8/8 |
| **Note**. The numbers indicate number of studies, except for the ‘cases/participants’ column. For example, in the ‘cancer type (RCC/KC)’ column, 5 studies for RCC and 6 studies for KC were included for ACEI.  **Abbreviations**. ACEI, angiotensin converting enzyme inhibitors; AHTN, antihypertensive medications; ARB, angiotensin receptor blockers; BB, beta-blockers; CCB, calcium-channel blockers; DU, diuretics; KC, kidney cancer; RCC, renal cell carcinoma  a Cohort studies included retrospective or prospective cohort, nested case-control, and case-cohort studies.  b Others included Europe, Middel East, Asia, and Australia.  c Studies that reported combined results for any classes of AHTN or non-DU were grouped as “Any AHTN”. | | | | | | | |

**Supplementary Table 5.** Pooled associations between AHTN and kidney cancer by pre-defined subgroups using all available estimates

|  | **n^a^** | **N^b^** | **Pooled RR 1^c^**  **(95% CI)** | **Pooled RR 2^c^ (95% CI)** | **P** |  | **n^a^** | **N^b^** | **Pooled RR 1^c^**  **(95% CI)** | **Pooled RR 2^c^  (95% CI)** | **P** |
| --- | --- | --- | --- | --- | --- | --- | --- | --- | --- | --- | --- |
| **ACEI** | | | | | | **CCB** | | | | | |
| **Outcome types** | | | | |  | **Outcome types** | | | | |  |
| **RCC** | 8 | 5 | **1.27 (1.14-1.42)** | 1.26 (0.89-1.78) | 0.91 | **RCC** | 8 | 5 | **1.37 (1.32-1.42)** | **1.37 (1.25-1.50)** | 0.82 |
| **KC** | 7 | 6 | 1.23 (0.83-1.82) | 1.17 (0.75-1.83) |  | **KC** | 12 | 9 | **1.41 (1.04-1.91)** | **1.41 (1.02-1.94)** |  |
| **AHTN assessment methods** | | | | | | **AHTN assessment methods** | | | | | |
| **Medical records** | 4 | 2 | 1.15 (0.15-9.06) | 1.15 (0.15-9.06) | 0.75 | **Medical records** | 4 | 2 | 1.34 (0.32-5.54) | 1.34 (0.32-5.54) | 0.82 |
| **Interview** | 11 | 9 | 1.29 (0.97-1.70) | 1.20 (0.87-1.64) |  | **Interview** | 16 | 12 | **1.41 (1.12-1.76)** | **1.40 (1.08-1.82)** |  |
| **Sex** |  |  |  |  |  | **Sex** |  |  |  |  |  |
| **Female** | 5 | 4 | 1.05 (0.68-1.62) | 1.04 (0.79-1.39) | 0.87 | **Female** | 7 | 5 | 1.76 (0.61-5.12) | 1.81 (0.38-8.48) | 0.34 |
| **Male** | 5 | 4 | 1.07 (0.77-1.48) | 1.06 (0.91-1.24) |  | **Male** | 7 | 5 | 1.26 (0.71-2.25) | 1.26 (0.62-2.53) |  |
| **Published year** | | | | | | **Published year** | | | |  |  |
| **Before 2000** | 5 | 3 | 1.86 (0.84-4.14) | 0.25 (0.02-2.66) | **0.02** | **Before 2000** | 7 | 5 | 1.39 (0.85-2.26) | 0.92 (0.29-2.94) | **0.02** |
| **After 2000** | 10 | 8 | 1.21 (0.95-1.54) | 1.20 (0.94-1.53) |  | **After 2000** | 13 | 9 | **1.40 (1.13-1.74)** | **1.41 (1.12-1.76)** |  |
| **Study design^d^** | | | |  |  | **Study design^d^** | | | |  |  |
| **Cohort** | 8 | 7 | 1.22 (0.90-1.64) | 1.20 (0.87-1.64) | 0.75 | **Cohort** | 13 | 10 | **1.40 (1.10-1.78)** | **1.40 (1.08-1.82)** | 0.82 |
| **Case-control** | 7 | 4 | 1.43 (0.45-4.47) | 1.15 (0.15-9.06) |  | **Case-control** | 7 | 4 | **1.37 (1.10-1.71)** | 1.34 (0.32-5.54) |  |
| **Estimate types** |  |  |  |  |  | **Estimate types** |  |  |  |  |  |
| **HR/IRR** | 2 | 2 | 1.31 (0.07-26.39) | 1.10 (0.58-2.10) | 0.35 | **HR/IRR** | 4 | 3 | 1.10 (0.73-1.65) | 1.10 (0.75-1.61) | 0.19 |
| **OR/RR** | 13 | 9 | 1.25 (0.98-1.61) | 1.19 (0.91-1.55) |  | **OR/RR** | 16 | 11 | **1.51 (1.25-1.81)** | **1.51 (1.23-1.85)** |  |
| **Quality scores** | | | | | | **Quality scores** | | | | | |
| **Low-moderate** | 5 | 4 | 1.57 (0.86-2.89) | 1.44 (0.08-26.57) | 0.29 | **Low-moderate** | 10 | 7 | 1.36 (0.78-2.36) | 1.36 (0.62-2.99) | 0.94 |
| **High** | 10 | 7 | 1.14 (0.88-1.47) | 1.13 (0.87-1.46) |  | **High** | 10 | 7 | **1.45 (1.07-1.98)** | **1.45 (1.01-2.07)** |  |
| **Geographical region** | | | | | | **Geographical region** | | | | | |
| **US/Canada** | 8 | 6 | 1.39 (0.96-2.00) | 1.23 (0.70-2.18) | 0.74 | **US/Canada** | 11 | 7 | 1.33 (0.97-1.82) | 1.32 (0.92-1.88) | 0.67 |
| **Europe/Middle East** | 4 | 3 | **1.27 (1.07-1.51)** | 0.94 (0.00-2954.75)^e^ |  | **Europe/Middle East** | 6 | 5 | **1.37 (1.10-1.69)** | 1.37 (0.93-2.00) |  |
| **Asia/Australia** | 3 | 2 | 1.05 (0.98-1.13) | 1.05 (0.98-1.13) |  | **Asia/Australia** | 3 | 2 | 1.71 (0.58-5.05) | 1.71 (0.58-5.05) |  |
| **ARB** | | | | | | **DU** | | | | | |
| **Outcome types** | | | | |  | **Outcome types** | | | | |  |
| **RCC** | 2 | 2 | **1.14 (1.00-1.30)** | **1.14 (1.04-1.25)** | 0.74 | **RCC** | 28 | 20 | **1.41 (1.04-1.91)** | **1.30 (1.12-1.51)** | 0.40 |
| **KC** | 7 | 6 | 1.15 (0.96-1.37) | 1.15 (0.96-1.37) |  | **KC** | 8 | 3 | **1.49 (1.07-2.07)** | **1.49 (1.07-2.07)** |  |
| **AHTN assessment methods** | | | | | | **AHTN assessment methods** | | | | | |
| **Medical records** | 0 | 0 | - | - | - | **Medical records** | 16 | 10 | **1.27 (1.03-1.57)** | 1.21 (0.97-1.51) | 0.21 |
| **Interview** | 9 | 8 | **1.15 (1.00-1.31)** | **1.15 (1.00-1.31)** |  | **Interview** | 20 | 13 | **1.51 (1.33-1.72)** | **1.45 (1.25-1.69)** |  |
| **Sex** |  |  |  |  |  | **Sex** |  |  |  |  |  |
| **Female** | 2 | 2 | 1.17 (0.45-3.03) | **1.25 (1.15-1.36)** | **0.02** | **Female** | 17 | 14 | **1.58 (1.24-2.01)** | **1.55 (1.04-2.30)** | 0.37 |
| **Male** | 2 | 2 | 1.17 (0.22-6.24) | **1.33 (1.23-1.43)** |  | **Male** | 15 | 12 | **1.51 (1.33-1.71)** | 1.20 (0.74-1.94) |  |
| **Published year** | | | | | | **Published year** | | | |  |  |
| **Before 2000** | 0 | 0 | - | - | - | **Before 2000** | 19 | 13 | **1.50 (1.26-1.80)** | **1.40 (1.15-1.71)** | 0.46 |
| **After 2000** | 9 | 8 | **1.15 (1.00-1.31)** | **1.15 (1.00-1.31)** |  | **After 2000** | 17 | 10 | **1.34 (1.06-1.69)** | **1.33 (1.02-1.73)** |  |
| **Study design^d^** | | | | | | **Study design^d^** | | | |  |  |
| **Cohort** | 9 | 8 | **1.15 (1.00-1.31)** | **1.15 (1.00-1.31)** | - | **Cohort** | 17 | 10 | **1.42 (1.22-1.65)** | **1.39 (1.16-1.67)** | 0.93 |
| **Case-control** | 0 | 0 | - | - |  | **Case-control** | 19 | 13 | **1.41 (1.14-1.74)** | **1.33 (1.07-1.66)** |  |
| **Estimate types** |  |  |  |  |  | **Estimate types** |  |  |  |  |  |
| **HR/IRR** | 5 | 5 | 1.07 (0.83-1.36) | 1.07 (0.83-1.36) | 0.13 | **HR/IRR** | 10 | 8 | **1.29 (1.13-1.47)** | **1.21 (1.07-1.36)** | 0.20 |
| **OR/RR** | 4 | 3 | 1.21 (0.93-1.57) | 1.21 (0.93-1.57) |  | **OR/RR** | 26 | 15 | **1.46 (1.26-1.69)** | **1.41 (1.20-1.67)** |  |
| **Quality scores** | | | | |  | **Quality scores** | | | | |  |
| **Low-moderate** | 2 | 2 | 1.04 (0.90-1.21) | 1.04 (0.96-1.13) | **<0.01** | **Low-moderate** | 13 | 9 | **1.32 (1.01-1.73)** | 1.27 (0.95-1.71) | 0.82 |
| **High** | 7 | 6 | **1.17 (1.00-1.37)** | **1.17 (1.00-1.37)** |  | **High** | 23 | 14 | **1.46 (1.30-1.64)** | **1.40 (1.21-1.62)** |  |
| **Geographical region** | | | | | | **Geographical region** | | | | | |
| **US/Canada** | 2 | 2 | 1.09 (0.69-1.71) | 1.09 (0.69-1.71) | 0.74 | **US/Canada** | 20 | 13 | **1.44 (1.17-1.78)** | **1.35 (1.11-1.65)** | 0.28 |
| **Europe/Middle East** | 2 | 2 | **1.14 (1.00-1.30)** | **1.14 (1.04-1.25)** |  | **Europe/Middle East** | 7 | 6 | **1.21 (1.01-1.45)** | 1.15 (0.84-1.57) |  |
| **Asia/Australia** | 5 | 4 | 1.19 (0.74-1.90) | 1.19 (0.74-1.90) |  | **Asia/Australia** | 9 | 4 | **1.47 (1.08-2.02)** | **1.47 (1.08-2.02)** |  |
| **BB** | | | | | | **Any AHTN** | | | | | |
| **Outcome types** | | | | |  | **Outcome types** | | | | |  |
| **RCC** | 11 | 8 | 1.19 (0.87-1.64) | 1.10 (0.89-1.35) | 0.87 | **RCC** | 18 | 13 | **1.43 (1.27-1.61)** | **1.28 (1.09-1.51)** | 0.20 |
| **KC** | 5 | 5 | 1.25 (0.87-1.80) | 1.09 (0.97-1.23) |  | **KC** | 5 | 3 | 1.96 (0.83-4.61) | 1.96 (0.83-4.61) |  |
| **AHTN assessment methods** | | | | | | **AHTN assessment methods** | | | | | |
| **Medical records** | 6 | 4 | 0.93 (0.45-1.92) | 0.98 (0.52-1.82) | 0.80 | **Medical records** | 14 | 9 | **1.56 (1.08-2.25)** | 1.42 (0.90-2.23) | 0.74 |
| **Interview** | 10 | 9 | **1.32 (1.07-1.64)** | **1.10 (1.00-1.20)** |  | **Interview** | 9 | 7 | **1.50 (1.34-1.69)** | **1.43 (1.17-1.76)** |  |
| **Sex** |  |  |  |  |  | **Sex** |  |  |  |  |  |
| **Female** | 6 | 5 | 1.15 (0.45-2.91) | 0.83 (0.15-4.70) | 0.87 | **Female** | 9 | 7 | **1.99 (1.58-2.51)** | 1.66 (0.13-20.48) | 0.97 |
| **Male** | 6 | 5 | 1.18 (0.76-1.83) | 1.03 (0.55-1.92) |  | **Male** | 8 | 6 | 1.58 (0.95-2.61) | 1.65 (0.00-1167.39)^e^ |  |
| **Published year** | | | | | | **Published year** | | | | | |
| **Before 2000** | 7 | 5 | 1.28 (0.58-2.83) | 0.70 (0.00-1917.96)^e^ | 0.99 | **Before 2000** | 13 | 9 | **1.41 (1.21-1.64)** | **1.34 (1.12-1.60)** | 0.37 |
| **After 2000** | 9 | 8 | **1.10 (1.01-1.20)** | **1.09 (1.04-1.15)** |  | **After 2000** | 10 | 7 | **1.75 (1.36-2.25)** | **1.70 (1.00-2.87)** |  |
| **Study design^d^** | | | | | | **Study design^d^** | | | |  |  |
| **Cohort** | 6 | 6 | 1.10 (0.97-1.24) | **1.10 (1.00-1.20)** | 0.80 | **Cohort** | 9 | 6 | **1.75 (1.21-2.53)** | 1.72 (0.95-3.11) | 0.13 |
| **Case-control** | 10 | 7 | 1.21 (0.78-1.88) | 0.98 (0.52-1.82) |  | **Case-control** | 14 | 10 | **1.38 (1.15-1.66)** | 1.24 (0.98-1.58) |  |
| **Estimate types** |  |  |  |  |  | **Estimate types** |  |  |  |  |  |
| **HR/IRR** | 4 | 4 | 1.30 (0.96-1.76) | 1.20 (0.80-1.79) | 0.32 | **HR/IRR** | 10 | 7 | **1.66 (1.20-2.30)** | 1.55 (0.94-2.56) | 0.32 |
| **OR/RR** | 12 | 9 | 1.19 (0.91-1.55) | **1.09 (1.03-1.16)** |  | **OR/RR** | 13 | 9 | **1.41 (1.14-1.74)** | 1.28 (0.94-1.73) |  |
| **Quality scores** | | | | | | **Quality scores** | | | | | |
| **Low-moderate** | 5 | 4 | 1.30 (0.63-2.67) | 1.09 (0.05-21.58) | 0.93 | **Low-moderate** | 12 | 8 | **1.75 (1.39-2.21)** | **1.73 (1.20-2.50)** | 0.23 |
| **High** | 11 | 9 | 1.10 (0.95-1.29) | **1.09 (1.03-1.16)** |  | **High** | 11 | 8 | **1.35 (1.11-1.65)** | 1.25 (0.96-1.61) |  |
| **Geographical region** | | | | | | **Geographical region** | | | | | |
| **US/Canada** | 8 | 6 | 1.28 (0.90-1.81) | 1.09 (0.94-1.25) | 0.63 | **US/Canada** | 9 | 6 | **1.64 (1.21-2.22)** | 1.40 (0.90-2.18) | 0.30 |
| **Europe/Middle East** | 5 | 4 | 0.98 (0.40-2.39) | 0.95 (0.31-2.94) |  | **Europe/Middle East** | 9 | 7 | **1.39 (1.14-1.69)** | 1.26 (0.96-1.64) |  |
| **Asia/Australia** | 3 | 3 | 1.34 (0.60-2.99) | 1.34 (0.60-2.99) |  | **Asia/Australia** | 5 | 3 | 1.86 (0.81-4.27) | 1.86 (0.81-4.27) |  |
| **Abbreviations**. ACEI, angiotensin converting enzyme inhibitors; AHTN, antihypertensive medications; ARB, angiotensin receptor blockers; BB, beta-blockers; CCB, calcium-channel blockers; DU, diuretics; HR, hazard ratio; IRR, incidence rate ratio; KC, kidney cancer; OR, odds, ratio; RCC, renal cell carcinoma; RR, risk ratio  ^a^ Number of estimates. We collected multiple estimates for the pre-defined subgroups from one study. For instance, multiple estimates according to sex or duration of AHTN exposure from one study were included. To include all these values, we employed the robust variance estimate method with random effect models.  ^b^ Number of studies.  ^c^ Pooled RR 1 includes all estimates, while Pooled RR 2 accounts for hypertension.  ^d^ Cohort studies included retrospective or prospective cohort, nested case-control, and case-cohort studies.  ^e^ Due to the small number of estimates among studies accounted for hypertension, a wide 95% confidence interval was observed. In each group, only two studies were included. | | | | | | | | | | |  |

**Supplementary Table 6.** Pooled estimates stratified by adjustment for smoking or body mass index in addition to hypertension

| **AHTN** | **Strata** | **No. of estimates** | **No. of studies** | **Pooled RR (95% CI)** | **I^2^ value** | **P** | **P for difference** |
| --- | --- | --- | --- | --- | --- | --- | --- |
| **ACEI** | Adjustment for HTN only | 6 | 5 | 1.20 (0.84-1.73) | 96.34 | 0.20 | 0.50 |
|  | Adjustment for HTN + smoking/BMI | 5 | 3 | 1.15 (0.62-2.12) | 0.00 | 0.23 |  |
| **ARB** | Adjustment for HTN only | 6 | 5 | **1.16 (1.01-1.34)** | 79.42 | **0.04** | 0.23 |
|  | Adjustment for HTN + smoking/BMI | 2 | 2 | 0.94 (0.38-2.33) | 0.00 | 0.56 |  |
| **BB** | Adjustment for HTN only | 4 | 4 | 1.10 (0.99-1.21) | 0.00 | 0.06 | 0.78 |
|  | Adjustment for HTN + smoking/BMI | 8 | 5 | 1.02 (0.70-1.50) | 35.20 | 0.87 |  |
| **CCB** | Adjustment for HTN only | 9 | 6 | **1.41 (1.05-1.88)** | 93.37 | **0.03** | 0.68 |
|  | Adjustment for HTN + smoking/BMI | 5 | 3 | 1.33 (0.90-1.98) | 0.00 | 0.07 |  |
| **DU** | Adjustment for HTN only | 12 | 6 | 1.40 (0.97-2.03) | 97.07 | 0.06 | 0.94 |
|  | Adjustment for HTN + smoking/BMI | 27 | 16 | **1.32 (1.11-1.57)** | 60.68 | **0.01** |  |
| **Any AHTN** | Adjustment for HTN only | 0 | 0 | NA |  |  | NA |
|  | Adjustment for HTN + smoking/BMI | 22 | 12 | **1.40 (1.13-1.75)** | 77.44 | **0.01** |  |
| **Abbreviation**. ACEI, angiotensin converting enzyme inhibitors; AHTN, antihypertensive medications; ARB, angiotensin receptor blockers; BB, beta-blockers; BMI, body mass index; CCB, calcium-channel blockers; DU, diuretics; HTN, hypertension; NA, not available; RR, relative risk | | | | | | | |

**Supplementary Table 7.** Sensitivity analyses for potential overlapping population issues

| **1) Sensitivity analysis excluding nine studies** | | | | | | | |
| --- | --- | --- | --- | --- | --- | --- | --- |
| **AHTN** | **No. of estimates** | **No. of studies** | **Pooled RR** | **Lower  95% CI** | **Upper  95% CI** | **I^2^ value** | **P** |
| ACEI | 10 | 8 | 1.42 | 1.05 | 1.91 | 86.98 | **0.03** |
| ARB | 4 | 4 | 1.10 | 0.99 | 1.24 | 15.16 | 0.07 |
| BB | 12 | 10 | 1.27 | 1.03 | 1.58 | 87.60 | **0.03** |
| CCB | 15 | 11 | 1.31 | 1.01 | 1.71 | 79.00 | **0.04** |
| DU | 26 | 19 | 1.45 | 1.28 | 1.63 | 71.22 | **<0.01** |
| Any AHTN | 20 | 14 | 1.51 | 1.27 | 1.80 | 62.52 | **<0.01** |
| **2) Sensitivity analysis excluding four studies** | | | | | | | |
| **AHTN** | **No. of estimates** | **No. of studies** | **Pooled RR** | **Lower  95% CI** | **Upper  95% CI** | **I^2^ value** | **P** |
| ACEI | 15 | 11 | 1.26 | 1.00 | 1.58 | 88.38 | **0.04** |
| ARB | 6 | 5 | 1.17 | 0.96 | 1.43 | 79.48 | 0.08 |
| BB | 16 | 13 | 1.24 | 1.02 | 1.45 | 70.30 | **0.03** |
| CCB | 20 | 14 | 1.40 | 1.15 | 1.71 | 82.69 | **<0.01** |
| DU | 35 | 22 | 1.41 | 1.26 | 1.59 | 90.35 | **<0.01** |
| Any AHTN | 23 | 16 | 1.52 | 1.30 | 1.77 | 76.76 | **<0.01** |
| To address overlapping population issues, such as within-population correlation between studies or within-study correlation between multiple estimates in our meta-analysis, we conducted two sensitivity analyses. We first identified nine overlapping studies using the same database within a similar study period and excluded them in the first sensitivity analysis: 1) Taiwan database: Chang, PY et al. (2015), Chen, LC et al. (2024), Chuang, YW et al. (2017); 2) US database: Colt, JS et al. (2011), Colt, JS et al. (2017); and 3) Korean database: Cho, IJ et al. (2021), Jeon, HL et al. (2022), Jung, MH et al. (2021), Kim, CS et al. (2020). In the second analysis, we retained one study per drug class, considered overlaps, and excluded studies with smaller patient populations or lower quality scores, resulting in the exclusion of four studies on diuretics and ARBs: 1) Chen, LC et al. (2024) (diuretics) due to a smaller patient population; 2) Cho, IJ et al. (2021) and Jung, MH et al. (2021) (ARB) due to smaller patient populations; and 3) Jeon, HL et al. (2022) (ARB) due to a lower quality score.  **Abbreviation**. ACEI, angiotensin converting enzyme inhibitors; AHTN, antihypertensive medications; ARB, angiotensin receptor blockers; BB, beta-blockers; BMI, body mass index; CCB, calcium-channel blockers; CI, confidence interval; DU, diuretics; HTN, hypertension; NA, not available; RR, relative risk | | | | | | | |

**Supplementary Table 8.** Leave-one-out sensitivity analysis for potential issues with highly influential studies

| **AHTN** | **No. of estimates** | **No. of studies** | **Pooled RR** | **Lower  95% CI** | **Upper  95% CI** | **I^2^ value** | **P** |
| --- | --- | --- | --- | --- | --- | --- | --- |
| ACEI | 14 | 10 | 1.26 | 0.95 | 1.66 | 89.00 | 0.08 |
| ARB | 8 | 7 | 1.15 | 0.96 | 1.37 | 75.58 | 0.10 |
| BB | 15 | 12 | 1.24 | 1.00 | 1.53 | 63.09 | 0.05 |
| CCB | 19 | 13 | 1.41 | 1.12 | 1.76 | 82.02 | **0.01** |
| DU | 35 | 22 | 1.42 | 1.26 | 1.60 | 89.13 | **<0.01** |
| Any AHTN | 23 | 16 | 1.52 | 1.30 | 1.77 | 76.76 | **<0.01** |
| **Abbreviation**. ACEI, angiotensin converting enzyme inhibitors; AHTN, antihypertensive medications; ARB, angiotensin receptor blockers; BB, beta-blockers; BMI, body mass index; CCB, calcium-channel blockers; CI, confidence interval; DU, diuretics; HTN, hypertension; NA, not available; RR, relative risk | | | | | | | |

**
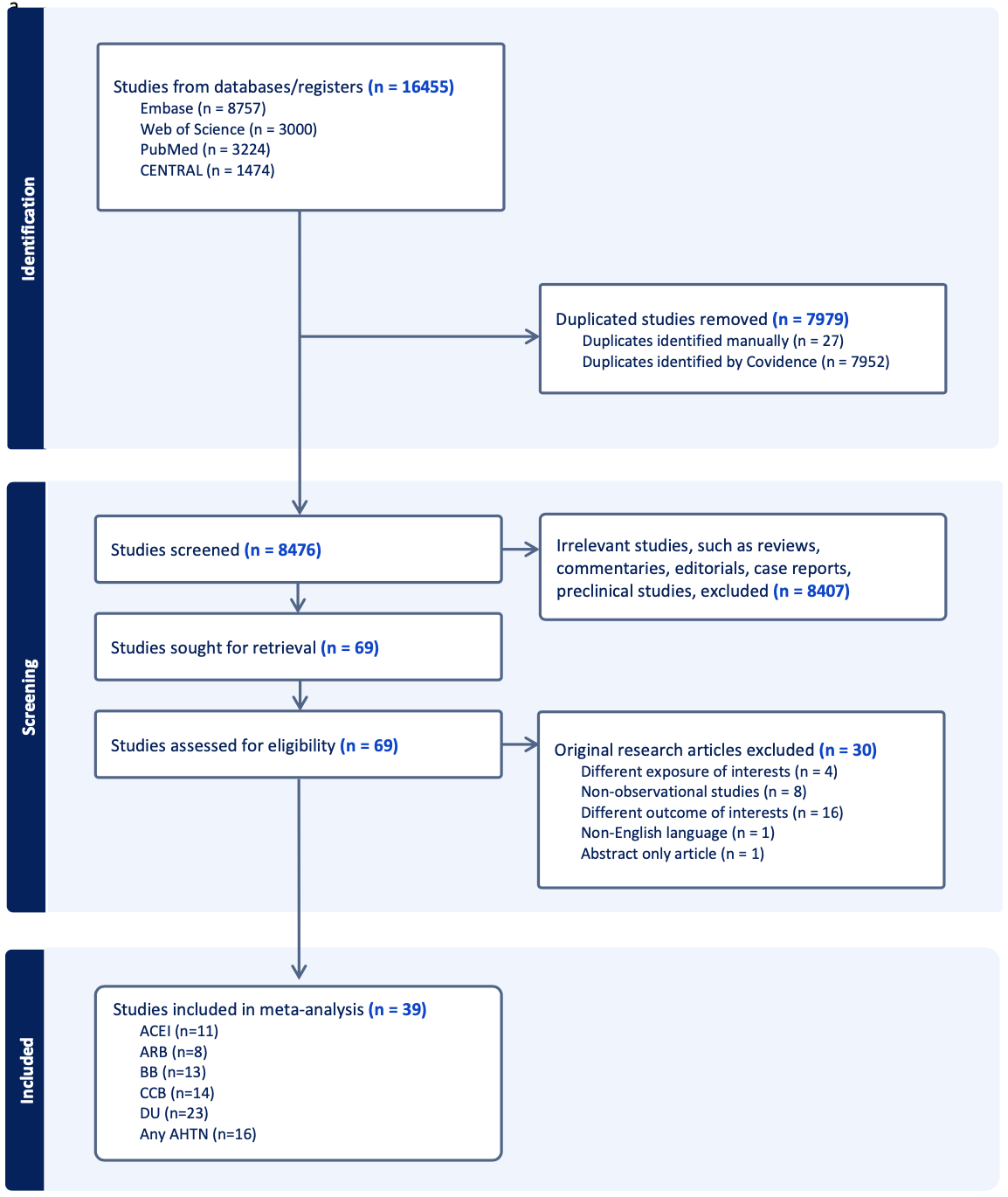
Supplementary Figure 1.** Flowchart of article screening and selection process along with inclusion and exclusion criteria

Supplementary Figure 1 Legend.

Comprehensive search strategies were established and implemented in PubMed, Embase, Web of Science, and Cochrane Library database through Jan 2025. In this meta-analysis, a total of 39 studies were included.

**Abbreviation**. ACEI, angiotensin converting enzyme inhibitors; AHTN, antihypertensive agents; ARB, angiotensin receptor blockers; BB, beta-blockers; CCB, calcium-channel blockers; DU, diuretics

**
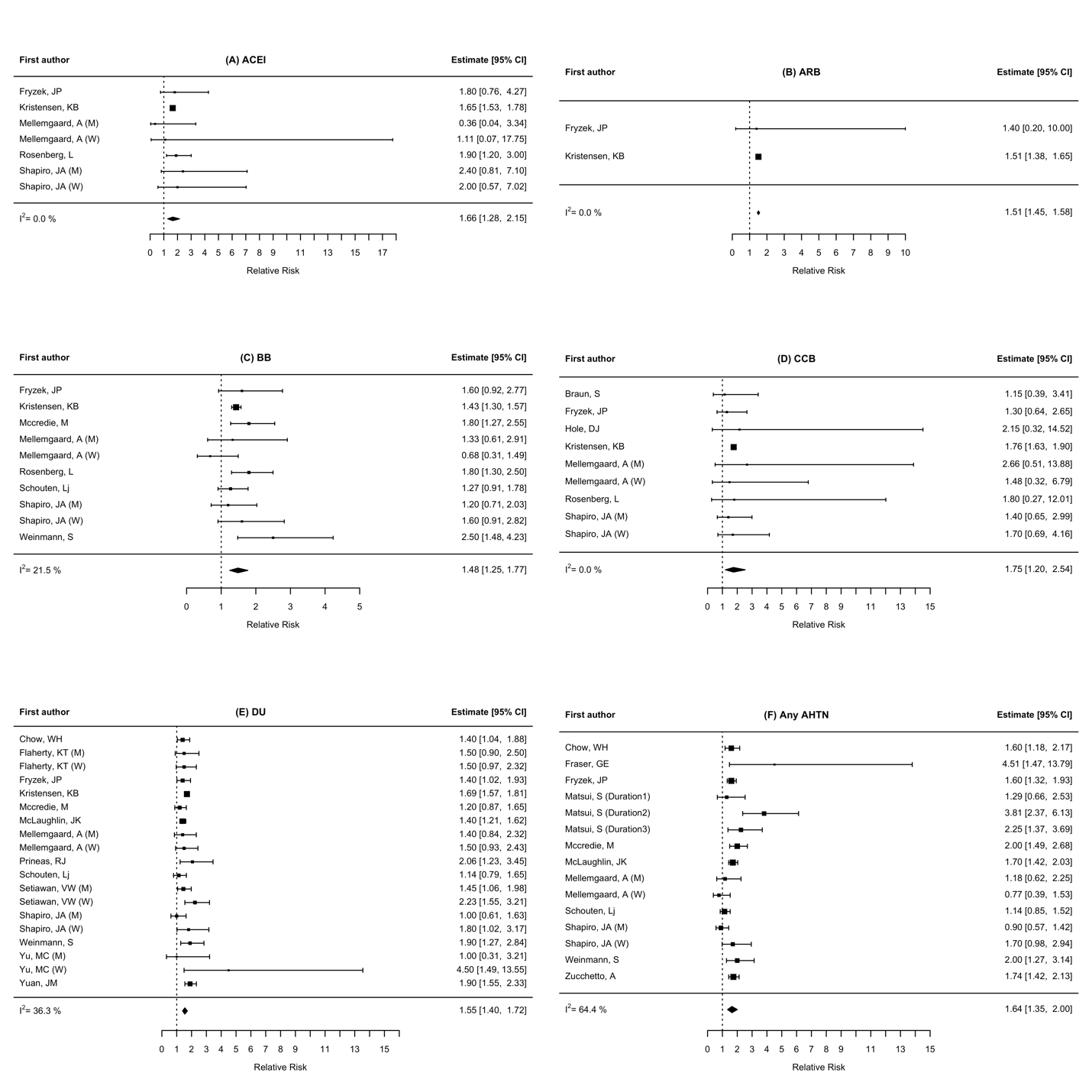
Supplementary Figure 2.** Forest plots of pooled estimates using all available estimates for the stratified analyses without accounting for hypertension

Supplementary Figure 2 Legend.

We observed that the pooled effects from estimates that did not account for hypertension showed stronger positive associations than those with accounting for hypertension across all classes of AHTN. Multiple estimates were chosen in several cases according to sex (men or women; M or W) or duration of medication use (Duration1-3).

**Abbreviation**. ACEI, angiotensin converting enzyme inhibitor; AHTN, antihypertensive medication; ARB, angiotensin receptor blocker; BB, beta-blocker; CCB, calcium-channel blocker; CI, confidence interval; DU, diuretic

**Supplementary Figure 3.** Bubble plots for dose-response relationships between antihypertensive medication use and kidney cancer risk


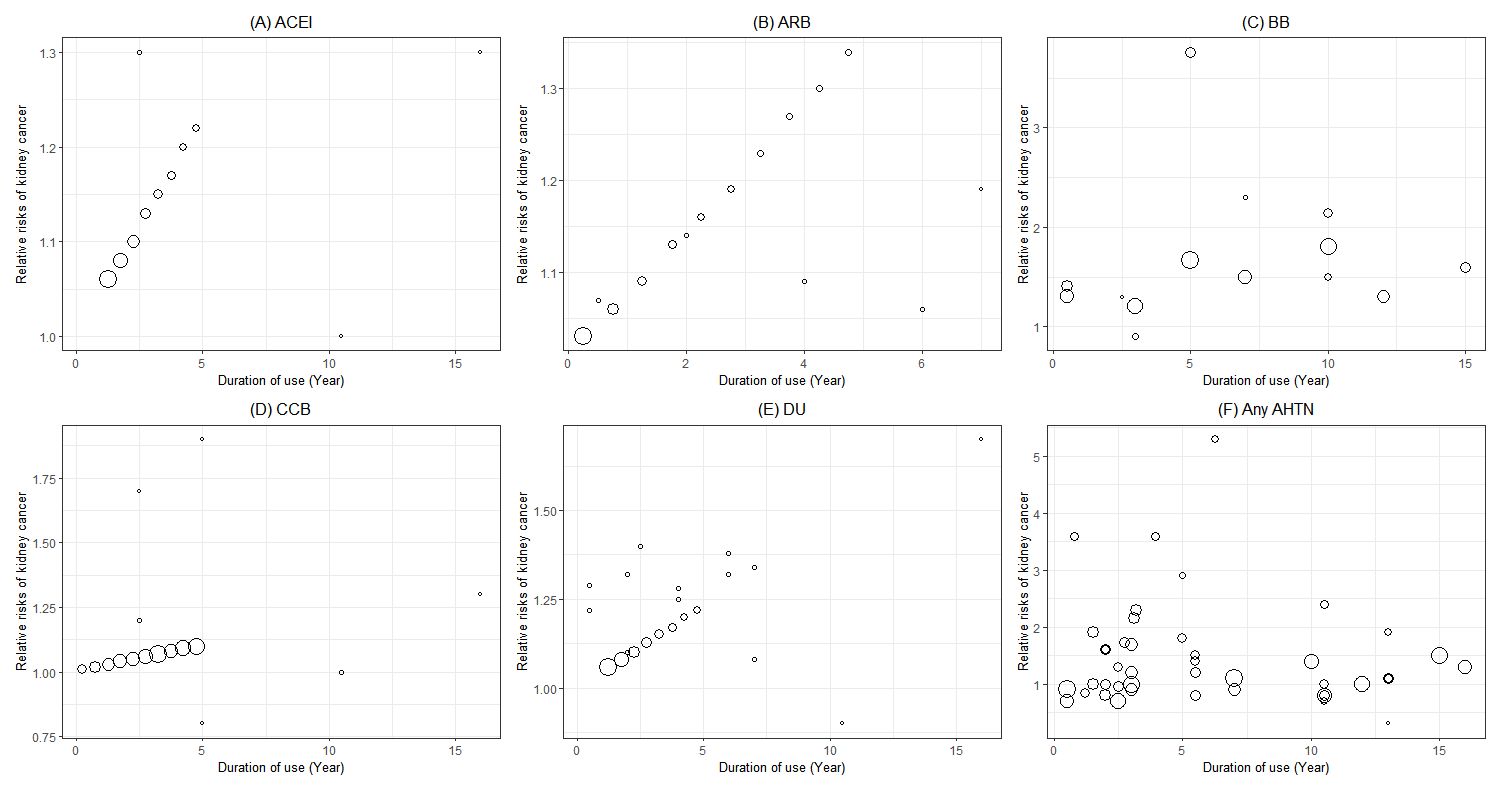


Supplementary Figure 3 Legend.

Antihypertensive medication use increased the risk of KC by 2-6% per additional year across all AHTN classes (A, B, C, D), while diuretics (E) and Any AHTN (F) showed no statistically significant associations. Relevant results are shown in Table 3. Bubble plots (a scatter plot weighted by standard error) to show the effect size (the natural log of relative risk) against the exposure duration. The size of each bubble reflected the study sample size, with larger bubbles indicating larger study sample size.

**Abbreviation**. ACEI, angiotensin converting enzyme inhibitors; ARB, angiotensin receptor blockers; BB, beta-blockers; CCB, calcium-channel blockers; DU, diuretics
